# The Resurgence of *Treponema pallidum* Infections and Reinfections during the COVID-19 Pandemic in Greece

**DOI:** 10.3390/ijerph21101283

**Published:** 2024-09-26

**Authors:** Andreas G. Tsantes, Panagiotis Toumasis, Aglaia Domouchtsidou, Electra Nicolaidou, Stefanos Bonovas, Alexander Stratigos, Athanasios Tsakris, Georgia Vrioni

**Affiliations:** 1 Department of Microbiology, Saint Savvas Oncology Hospital, 11522 Athens, Greece; andreas.tsantes@yahoo.com (A.G.T.); ldomouchtsidou@gmail.com (A.D.); 2 Department of Microbiology, Medical School, National and Kapodistrian University of Athens, 11527 Athens, Greece; toumasispanagis@gmail.com (P.T.); atsakris@med.uoa.gr (A.T.); 3 1st Department of Dermatology and Venereology, Andreas Sygros Hospital, National and Kapodistrian University of Athens, 11527 Athens, Greece; electra.nicol@gmail.com (E.N.); alstrat2@gmail.com (A.S.); 4 Department of Biomedical Sciences, Humanitas University, 20072 Milan, Italy; 5 IRCCS Humanitas Research Hospital, 20089 Milan, Italy

**Keywords:** syphilis, COVID-19, sexually transmitted infections, reinfection

## Abstract

The beginning of the COVID-19 pandemic was marked by a sharp decline in syphilis infections in many countries worldwide, including Greece. However, a resurgence of positive cases started to appear in the second half of 2020. The aim of this study was to explore the impact of the pandemic on the incidence of new syphilis infections and reinfections and analyze the sociodemographic characteristics associated with recurrent episodes. We analyzed medical records from a 14-month period after the beginning of the pandemic and compared them with the respective period before the start of the pandemic. Our participants consisted mainly of men, with a median age of 43 years, homosexual orientation, and higher education. During COVID-19, more HIV patients presented for syphilis testing (38.0% vs. 34.6%, *p* = 0.025). Overall, we observed almost a two-fold increase in positive syphilis cases during COVID-19 (21.1% vs. 12.4%, *p* < 0.001), with new infections rising from 8.4% to 13.2% and reinfections from 4.0% to 7.9%. Multivariable logistic regression analysis revealed that the COVID-19 pandemic, among different parameters (such as age, gender, sexual orientation, HIV status, and educational level), was the only factor associated with higher positive syphilis rates (OR 1.47, 95% CI: 1.07–2.01, *p* = 0.003). Our results highlight the need to ensure enhanced prevention and undisrupted healthcare services, with a focus on future pandemics.

## 1. Introduction

On 11 May 2020, the World Health Organization declared COVID-19 a pandemic and recommended that countries take action to restrain the virus [1]. In response, the Greek government implemented two nationwide lockdown programs of social distancing, self-isolation for contacts, and prohibition of public gatherings. The first lockdown began on 23 March 2020 and lasted until 4 May 2020, and the second lockdown began on 7 November 2020 and lasted until 14 May 2021.

Syphilis is a systemic disease caused by the bacterium *Treponema pallidum* subspecies *pallidum*. It is mainly transmitted through sexual contact, leading to clinical symptoms that are divided into four different stages (primary, secondary, latent, and tertiary phases) [2]. Due to the atypical symptoms in the primary and secondary phases, which mostly resolve after a period of time without treatment, the transmission of syphilis may go undetected, unless systematic prevention and surveillance programs are implemented. In 2022, a crude notification rate of 8.5 syphilis cases per 100,000 population was reported in 29 European countries, indicating a 34% increase compared to 2021 and a 41% increase compared to 2018. A steady increase in the syphilis notification rate has been observed over the past decade. In Greece, the crude notification rate of syphilis cases was 8.3 per 100,000 population in 2022, which is in line with the mean syphilis rate in Europe [3]. The COVID-19 pandemic has had a significant impact on many aspects of healthcare worldwide, including the diagnosis and management of sexually transmitted infections (STIs) such as syphilis.

While measures to prevent the spread of SARS-CoV-2, such as lockdowns, have been effective in reducing the transmission of the virus, they have also led to changes in the epidemiology of STIs worldwide. Several studies conducted in countries around the world showed that the COVID-19 pandemic initially resulted in a decline in the number of people being diagnosed with syphilis [4,5,6,7]. This decrease was believed to be a consequence of social distancing measures and limited access to healthcare services during the pandemic. In line with this, a study performed in Greece from 1 March 2020 to 30 October 2020 also observed that newly diagnosed cases of syphilis were reduced by 10% in comparison with the respective numbers of the same period in 2019 [8]. However, shortly after the beginning of the pandemic, in the second half of 2020, reports from different countries started to show a resurgence of syphilis cases [9,10], probably indicating an underdiagnosis of syphilis transmission in the first months after the COVID outbreak.

To shed light on the situation, the present study was conducted in order to investigate the impact of the COVID-19 pandemic and the respective isolation measures on the emergence of new infections and reinfections caused by *T. pallidum* in Greece. Moreover, we aimed to investigate whether COVID-19 was associated with any changes in the characteristics and demographics of patients with syphilis.

## 2. Materials and Methods

### 2.1. Study Population

A retrospective study was conducted including individuals who presented to the “National Reference Center for Sexually Transmitted Infections and AIDS” at “Andreas Sygros Hospital for Venereal & Dermatological Diseases” in Athens between 1 January 2019 and 31 May 2021 for serological syphilis testing, either due to a clinical suspicion of syphilis or as part of routine monitoring (follow-up). Patients with persistent syphilis infections were excluded. Patients’ records were reviewed from 14 months before the start of the pandemic to 14 months after the start of the pandemic. The breakpoint was March 2020, when the World Health Organization declared COVID-19 a global emergency and the first nationwide lockdown was introduced in Greece. These individuals were either external patients of the sexually transmitted diseases clinic or patients being followed up in the hospital’s specialized infectious diseases unit. The study was approved by the Institutional Review Board of the “Andreas Sygros” hospital (approval number 121/19 March 2024).

This study provides a secondary analysis of the impact of COVID-19 disease on syphilis cases, since a previous study from our department provided data regarding the incidence of new syphilis cases in a population of 3600 tested individuals before the COVID-19 period and 2905 patients during the COVID-19 pandemic, showing that the incidence of new syphilis cases increased from 10.5% to 21% during COVID-19 [11]. The current study evaluated the impact of the COVID-19 pandemic on the incidence of syphilis reinfections, as well as providing additional data on the characteristics and demographics of patients with infections before and during the pandemic.

### 2.2. Laboratory Testing

Serological testing for syphilis included a non-treponemal test, namely, the Venereal Disease Research Laboratory (VDRL) test [VDRL Antigen MR, Linear Chemicals, Barcelona, Spain], and two treponemal tests, namely, the *T. pallidum* passive particle agglutination (TP-PA) assay [Serodia-TP-PA, Fujirebio Inc., Tokyo, Japan] and an enzyme immunoassay (EIA) for the detection of immunoglobulin M (IgM) and immunoglobulin G (IgG) antibodies to *T. pallidum* [Treponema IgG and IgM, Delta Biologicals, Pomezia, Italy].

Based on the results of the above serological tests, a conclusion was drawn whereby each case was assigned to one of the following four groups: serological profiles compatible with new infections with *T. pallidum*, serological profiles compatible with reinfections with *T. pallidum*, serological profiles compatible with past/treated infections with *T. pallidum* and serological profiles compatible with no infection with *T. pallidum*. The above process was based on the latest guidelines provided by the Centers for Disease Control and Prevention (Supplementary Material) [12,13,14,15,16].

### 2.3. Statistical Analysis

The statistical analysis included descriptive statistics of the study population regarding demographics. The distribution of data was evaluated for normality through the Shapiro–Wilk test. These variables were compared between tested individuals before and during the COVID-19 period using the nonparametric Wilcoxon rank-sum test and the chi-square test when appropriate. The percentages of positive and negative syphilis results were compared between tested individuals before and during the COVID-19 period using the chi-square test. Moreover, continuous and categorical variables were compared using the nonparametric Wilcoxon rank-sum test and the chi-square test in positive for syphilis patients before and during the COVID-19 period. In order to investigate whether the COVID-19 period was independently associated with a higher rate of positive syphilis results, a multivariable logistic regression analysis was performed with a positive syphilis result as the dependent variable, and age, gender, sexual orientation, HIV status, education level, and COVID-19 period as the independent variables. The statistical analysis was performed using the Stata 15.0 software (Stata Corp., College Station, TX, USA), while a *p*-value lower than 0.05 indicated statistical significance for all tests.

## 3. Results

Overall, 4849 individuals were tested during the study period; 2069 individuals were tested for syphilis before the COVID-19 period, and 2780 individuals were tested during the COVID-19 period (Table 1). Three patients with persistent infections before the COVID-19 period and six patients during the COVID-19 period were excluded.

**Figure 1:**
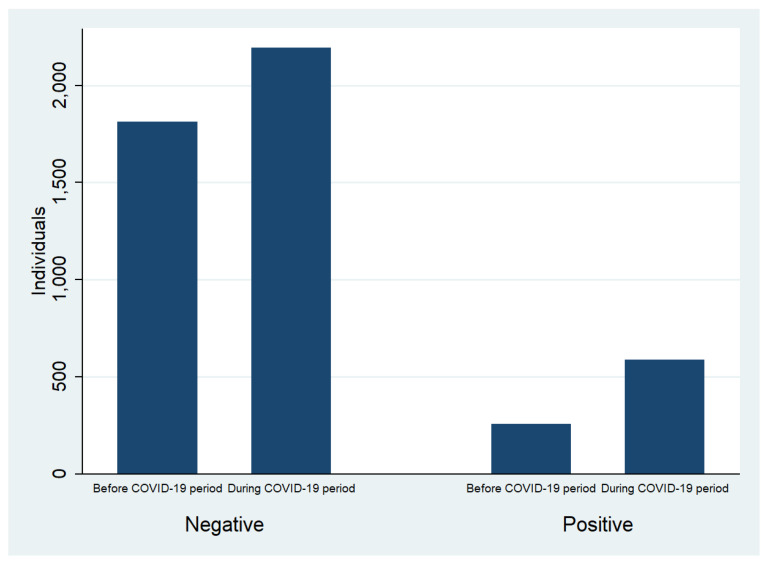
Positive and negative syphilis cases before and during the COVID-19 period.

Regarding the infection status of the tested population for *T. pallidum*, there were 256 individuals with serological profiles compatible with syphilis infections (new infections and reinfections) prior to the COVID-19 period, while there were 587 individuals with serological profiles compatible with syphilis infections during the COVID-19 period (Figure 1). The overall incidence was 12.4% (95% confidence interval [CI]: 10.9–13.9) prior to the COVID-19 period and 21.1% (95% CI: 19.4–22.8) during the COVID-19 period.

**Figure 2:**
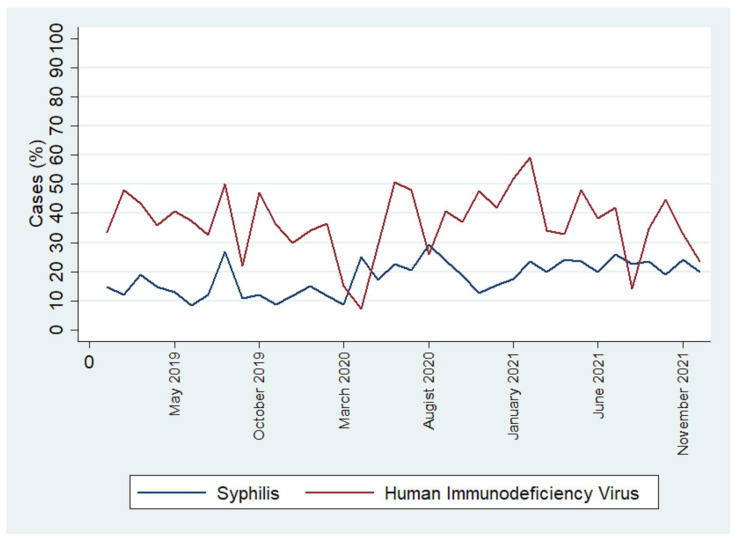
Incidence rates of syphilis and HIV in the population over the study period.

The incidence was higher during the COVID-19 period (*p* < 0.001), indicating that the COVID-19 period was associated with an increase in syphilis cases (Table 2). Moreover, the incidence of new infections during the COVID-19 period (13.2%, 95% CI: 11.9–14.6) was higher than that before the COVID-19 period (8.4%, 95% CI: 7.2–9.7; *p* < 0.001), while the incidence of reinfections during the COVID-19 period (7.9%, 95% CI: 6.8–8.9) was also higher than that before the COVID-19 period (4.0%, 95% CI: 3.1–4.9; *p* < 0.00; Figure 2). A detailed time-based analysis did not reveal any specific intervals during the study period that could have influenced the syphilis rate. Specifically, the impact of COVID-19 lockdowns was assessed by comparing the syphilis incidence during lockdown periods with that when restrictions were lifted, but no significant difference was observed (21.8% vs. 19.2%, *p* = 0.13). Additionally, a comparison of syphilis incidence between the late pandemic period (May 2021–December 2021) and the early pandemic period (March 2020–March 2021) showed no substantial difference (20.1% vs. 22.3%, *p* = 0.16).

Interestingly, although the percentage of individuals with serological profiles compatible with no infection decreased during the COVID-19 period compared to before (49.9% vs. 60.8%, *p* < 0.001), the percentage of individuals with serological profiles compatible with past infections increased during the COVID-19 period (30.0% vs. 26.8%, *p* = 0.048; Table 2).

Regarding those patients with serological profiles compatible with syphilis infections (new infections and reinfections), there were no differences in their characteristics before and during the COVID-19 period (Table 3). Specifically, the age of patients with positive results was comparable before and during the COVID-19 period (medians: 44 vs. 42 years, *p* = 0.20), as were sexual orientation (bisexual: 78.5% vs. 80.4%, *p* = 0.70), gender (*p* = 0.39), HIV status (*p* = 0.68), and education level (*p* = 0.55), indicating that the characteristics of patients positive for syphilis did not change during the COVID-19 period.

Finally, the findings of multivariable logistic regression analysis further supported an association between the COVID-19 period and syphilis infections (Table 4). Specifically, the COVID-19 period was associated with a higher rate of positive results for syphilis (odds ratio [OR] 1.47, 95% CI: 1.07–2.01; *p* = 0.003), while a positive syphilis serological profile was not associated with gender (OR 0.56, 95% CI: 0.19–1.62; *p* = 0.29), age (OR 0.99, 95% CI: 0.97–1.00; *p* = 0.25), sexual orientation (OR 1.35, 95% CI: 0.90–2.03; *p* = 0.14), HIV status (OR 0.72, 95% CI: 0.52–1.00; *p* = 0.055), or education level (OR 1.07, 95% CI: 0.82–1.38, *p* = 0.53).

## 4. Discussion

Although measures to prevent the spread of SARS-CoV-2, such as isolation and lockdowns, were effective in decreasing the spread of the virus, they also had a great impact on the epidemiology of STIs worldwide. Several reasons for this have been reported. In the current study, we compared the incidence of syphilis cases treated in our hospital during the first 14 months of the pandemic to that in a similar period before the outbreak of COVID-19. Our findings indicate that the total number of positive cases increased, including new infections, while reinfections showed almost a two-fold increase.

It has been reported that restricted access to health services and an unwillingness to go in for testing for fear of contracting COVID-19 may have resulted in the underdiagnosis of STIs during the pandemic [17,18]. However, as shown in our study, more people presented for syphilis serological testing during the COVID-19 period, with this trend being more apparent in the subgroup of HIV-positive patients. This finding could be attributed to the fact that our cohort of participants included outpatients for whom the benefit of testing could possibly overcome any concern about contracting COVID-19. Another reason may be the fact that our hospital specializes exclusively in cutaneous and venereal diseases, without admitting any COVID-19 patients. Therefore, it is possible that more individuals were tested in our hospital due to their avoidance of other hospitals where COVID-19 patients were being treated.

The beginning of the COVID-19 pandemic was marked by a significant reduction in STIs in many countries [4,5,6,19], raising questions as to whether this was real or due to decreased testing and limited access to sexual health services during the first lockdowns [7,17,18]. However, the decrease in the diagnoses of syphilis was short-lived. The initial decrease in early syphilis was followed by a significant increase during the second half of 2020. A study from the Czech Republic recorded a resurgence of syphilis cases reported between March 2020 and February 2021 [9]. In Cuba, the first quarantine measures resulted in reduced incidence rates of syphilis, but a rebound to former rates appeared with the relaxation of restrictions [10]. Our results are also in line with other studies reporting an increase in syphilis cases during 2020–2021 [20,21]. An increase in cases in late 2020, when the first restrictions were temporarily lifted, may indicate that more people sought care when it was available [20] and that the limited access to healthcare in the first half of 2020 led to markedly disrupted prevention and care of STIs [22]. Since early syphilis does not manifest with severe symptoms, it is possible that those who were infected went undiagnosed for a prolonged period, transmitting it to their partners.

Interestingly, our study revealed that the incidence of reinfections also increased. Several studies analyzing epidemiological and clinical factors contributing to recurrent episodes of syphilis found that male sex, HIV coinfection, homosexual/bisexual orientation, and the absence of syphilis symptoms at the time of the diagnosis were associated with a higher prevalence of reinfections [23,24,25]. Moreover, the disruption of preventive services during the COVID-19 pandemic, including the slowing of STI control programs, may also be associated with the increased syphilis rates observed during this period [26]. The group of patients with syphilis during COVID-19 consisted mostly of men (95.2%) with a homosexual orientation (80.4%), while our results also indicate a similar rate of HIV coinfection in patients with syphilis before and during the pandemic (36.4% vs. 34.9%). As opposed to the similar rate of HIV prevalence among patients with syphilis before and during the pandemic, another study performed in Greece showed that new diagnoses of syphilis among people with HIV (mainly men who have sex with men—MSM) dropped at the beginning of the first lockdown but increased in subsequent months [27]. In Croatia, the syphilis rate increased by 91.4% in men with HIV between 2019 and 2020, with a higher incidence in the MSM subpopulation [16].

Since syphilis is a sexually transmitted disease and its incidence corresponds to increased sexual activity, a drop in syphilis incidence would be expected during the pandemic, when the population was in confinement. However, the increased incidence that was observed during the pandemic could be attributed not to increased sexual activity but to changes in sexual practice on a behavioral level. During the pandemic, due to the restrictions imposed on social interactions, sexual behavior and expression were immensely modified [28,29]. Online partner seeking, engagement in risky sexual behaviors, an increased incidence of binge drinking, and greater recreational drug use or unprotected sex are some of the reasons that may have led to recurrent syphilis infections [30,31,32]. Such changes in sexual practice in the Greek population should be investigated as potential causes of the observed increase in the syphilis rate that was evident in our study. A survey that was conducted among gay, bisexual, and MSM in the UK revealed increased sexual activity from February to May 2020 and a strong connection between high-risk sexual behavior and the use of substances or excessive alcohol consumption [33]. The use of pre-exposure prophylaxis (PrEP) for the prevention of HIV transmission could also have played a role, but since PrEP is still not officially available in Greece and people can have access to these medications only on the online market, the impact of PrEP cannot be documented in our country.

Studies from other countries suggest that the dynamics changed as the pandemic unfolded, with the effect of the first wave on social and sexual activities being different compared to the subsequent waves [18]. Our data encompass the entire duration of the COVID-19 pandemic in Greece, and a time-based analysis did not identify any specific intervals during the study period that may have influenced the rise in syphilis rates. Specifically, our analysis indicates that lockdown periods were not associated with a higher syphilis incidence compared to periods when restrictions were lifted. Moreover, the syphilis incidence rates during the later phase of the pandemic (May 2021–December 2021) were similar to those observed during the initial phase (March 2020–March 2021).

This is a secondary analysis of the impact of COVID-19 disease on syphilis cases, since a previous study provided data regarding the incidence of new syphilis cases in a larger population compared to the current study (6505 vs. 4849 individuals) [11]. However, in our study, we provided detailed criteria for the laboratory diagnosis of syphilis (new infections, past/treated infections, reinfections), which enabled us to evaluate the reinfection rate before and during the COVID-19 pandemic, as opposed to the previous study in the Greek population. Moreover, there was a certain number of patients with missing data regarding several parameters, such as sexual orientation and education level. This can lead to biased results regarding the evaluation of the association between changes in syphilis incidence and the COVID-19 pandemic. Another limitation of our study is that an evaluation of changes in sexual practice on a behavioral level that resulted in the increased incidence of new syphilis infections and reinfections was not performed. Data on specific sexual practices such as condom use or risky behaviors such as chemsex or the use of dating sites to find new partners were not collected. Future studies including data and questionnaires focused on changes in sexual behavior and their impact on syphilis incidence would be valuable.

## 5. Conclusions

Our findings indicate that the total number of syphilis cases increased during the first year of the COVID-19 pandemic, with reinfections showing an almost a two-fold increase. This is in line with similar studies from the rest of the world, showing an increase in syphilis cases after a short period following the breakout of the COVID-19 pandemic. Interestingly, although this worldwide rise in STDs has been attributed to restricted access to health services and an unwillingness to go in for testing for fear of contracting COVID-19, a higher number of people in our study presented for syphilis serological testing during the COVID-19 period. However, this may be attributed to the specific nature of our hospital, which specializes exclusively in venereal diseases and does not admit COVID-19 patients. As a result, individuals may have chosen to seek testing at our facility rather than at other hospitals where they could potentially encounter COVID-19 patients. These findings indicate that, although the reason for the increased incidence of syphilis in the Greek population during the isolation period could be attributed to changes in sexual behavior, this should be investigated in future studies. The recent pandemic has shown that, in view of future pandemics, there is a strong need to ensure undisrupted prevention, systematic testing, and flexible care services, especially for asymptomatic STIs such as syphilis.

## Figures and Tables

**Table 1:** Characteristics of individuals who presented for serological syphilis testing before and during the COVID-19 period.

Parameters	Before COVID-19 Period (n = 2069)	During COVID-19 Period (n = 2780)	*p*-Value
Gender			<0.001
Male	1831 (88.5)	2554 (91.9)	
Female	234 (11.3)	221 (7.9)	
Transgender female	4 (0.2)	5 (0.2)	
Age (years)	43.0 (34.5–52.0)	43.0 (34.0–52.0)	0.66
HIV-positive *	606 (34.6)	925 (38.0)	0.025
Sexual orientation **			0.055
Homosexual/bisexual	484 (72.8)	694 (77.1)	
Heterosexual	181 (27.2)	207 (22.9)	
Education level ***			0.57
Lower	59 (9.1)	83 (9.4)	
Middle	206 (31.7)	257 (29.2)	
Higher	384 (59.2)	539 (61.4)	

Data are presented as medians and interquartile ranges (IQRs) or as frequencies (percentages). The nonparametric Wilcoxon rank-sum test and the chi-square test were used for comparisons between the 2 groups. * A total of 1747 participants before the COVID-19 period and 2430 participants during the COVID-19 period. ** A total of 665 participants before the COVID-19 period and 901 participants during the COVID-19 period responded regarding their sexual orientation. *** A total of 649 participants before the COVID-19 period and 879 participants during the COVID-19 period responded regarding their education level.

**Table 2:** Syphilis serological profiles of tested individuals before and during the COVID-19 period.

Serological Profile	Before COVID-19 Period (n = 2069)		During COVID-19 Period (n = 2780)		*p*-Value
	Number of Cases	Incidence (95% Confidence Interval)	Number of Cases	Incidence (95% Confidence Interval)	
Positive	256	12.4% (10.9–13.9)	587	21.1% (19.4–22.8)	<0.001
New infections	174	8.4% (7.2–9.7)	368	13.2% (11.9–14.6)	<0.001
Reinfections	82	4.0% (3.1–4.9)	219	7.9% (6.8–8.9)	<0.001
Negative	1813	87.6% (83.6–91.7)	2193	78.9% (75.6–82.2)	<0.001
Old infections	554	26.8% (24.5–29.1)	806	30.0% (27.0–31.0)	0.048
No infection	1259	60.8% (57.5–64.3)	1387	49.9% (47.3–52.5)	<0.001

The chi-square test was used for comparisons between the 2 groups.

**Table 3:** Characteristics of patients positive for syphilis (serological profile compatible with new infection and reinfection).

Parameters	Before COVID-19 (n = 256)	During COVID-19 (n = 587)	*p*-Value
Gender			0.39
Male	238 (93.0)	559 (95.2)	
Female	17 (6.6)	27 (4.6)	
Transgender female	1 (0.4)	1 (0.2)	
Age (years)	44 (35–51)	42 (34–50)	0.20
Sexual orientation *			0.70
Homosexual/bisexual	73 (78.5)	156 (80.4)	
Heterosexual	20 (21.5)	38 (19.6)	
HIV-positive **	77 (36.4)	177 (34.9)	0.68
Education level ***			0.55
Lower	10 (10.1)	13 (6.5)	
Middle	31 (31.3)	66 (33.3)	
Higher	58 (58.6)	119 (60.2)	

Data are presented as medians and interquartile ranges (IQRs) or as frequencies (percentages). The nonparametric Wilcoxon rank-sum test and the chi-square test were used for comparisons between the 2 groups. * A total of 93 participants before the COVID-19 period and 194 participants during the COVID-19 period responded regarding their sexual orientation. ** A total of 211 participants before the COVID-19 period and 507 participants during the COVID-19 period had available results regarding HIV status. *** A total of 99 participants before the COVID-19 period and 198 participants during the COVID-19 period responded regarding their education level.

**Table 4:** Multivariable logistic regression investigating the independent association between syphilis and COVID-19, adjusted for gender, age, sexual orientation, HIV status, and education level.

Variables	Positive Syphilis Serological Profile		
	Adjusted OR	95% CI	*p*-Value
Gender	0.56	0.19–1.62	0.29
Age	0.99	0.97–1.00	0.25
Sexual orientation	1.35	0.90–2.03	0.14
Education level	1.07	0.82–1.38	0.53
COVID-19 period	1.47	1.07–2.01	0.003
HIV	0.72	0.52–1.00	0.055

Abbreviations: OR, odds ratio; CI, confidence interval.

## Data Availability

The data presented in this study are available on request from the corresponding author.

## Supplementary Materials

The following supporting information can be downloaded at: https://www.mdpi.com/article/10.3390/ijerph21101283/s1, Definition of infections.

## References

[1] Kaur T., Mahajan M., Mahajan B.B. (2023). Syphilis resurgence: Exploring the impact of COVID-19 pandemic. Indian J. Sex. Transm. Dis. AIDS.

[2] O’Byrne P., MacPherson P. (2019). Syphilis. BMJ.

[3] Annual Epidemiological Report 2022—Syphilis. https://www.ecdc.europa.eu/sites/default/files/documents/SYPH_AER_2022_Report_0.pdf.

[4] de Miguel Buckley R., Trigo E., de la Calle-Prieto F., Arsuaga M., Díaz-Menéndez M. (2020). Social distancing to combat COVID-19 led to a marked decrease in food-borne infections and sexually transmitted diseases in Spain. J. Travel Med..

[5] Chia C.C., Chao C.M., Lai C.C. (2021). Diagnoses of syphilis and HIV infection during the COVID-19 pandemic in Taiwan. Sex. Transm. Infect..

[6] Latini A., Magri F., Donà M.G., Giuliani M., Cristaudo A., Zaccarelli M. (2021). Is COVID-19 affecting the epidemiology of STIs? The experience of syphilis in Rome. Sex. Transm. Infect..

[7] Sentís A., Prats-Uribe A., López-Corbeto E., Montoro-Fernandez M., Nomah D.K., de Olalla P.G., Mercuriali L., Borrell N., Guadalupe-Fernández V., Reyes-Urueña J. (2021). The impact of the COVID-19 pandemic on Sexually Transmitted Infections surveillance data: Incidence drop or artefact?. BMC Public Health.

[8] Apalla Z., Lallas A., Mastraftsi S., Giannoukos A., Noukari D., Goula M., Kalantzi P., Zapridou M., Lallas K., Kyrgidis A. (2022). Impact of COVID-19 pandemic on STIs in Greece. Sex. Transm. Infect..

[9] Bížová B., Rob F., Třešňák Hercogová J. (2022). Increase of early syphilis cases during the COVID-19 pandemic in the Czech Republic. Sex. Transm. Infect..

[10] Rodríguez I., Hernández Y. (2021). Sexually transmitted diseases during the COVID-19 pandemic: A focus on syphilis and gonorrhoea in Cuba. Public Health Pract..

[11] Nicolaidou E., Fouseki K., Paparizos V., Kotsafti O., Vasalou V., Daskalakis E., Lakoumentas J., Giannoukos A., Emmanouil G., Kapranou R. (2024). A sharp increase in early syphilis cases in a referral hospital in Athens, Greece, 2 years into the COVID-19 pandemic. J. Eur. Acad. Dermatol. Venereol..

[12] Papp J.R., Park I.U., Fakile Y., Pereira L., Pillay A., Bolan G.A. (2024). CDC Laboratory Recommendations for Syphilis Testing, United States, 2024. MMWR Recomm. Rep..

[13] Marchese V., Tiecco G., Storti S., Degli Antoni M., Calza S., Gulletta M., Viola F., Focà E., Matteelli A., Castelli F. (2022). Syphilis Infections, Reinfections and Serological Response in a Large Italian Sexually Transmitted Disease Centre: A Monocentric Retrospective Study. J. Clin. Med..

[14] Boog G.H.P., Lopes J.V.Z., Mahler J.V., Solti M., Kawahara L.T., Teng A.K., Munhoz J.V.T., Levin A.S. (2021). Diagnostic tools for neurosyphilis: A systematic review. BMC Infect. Dis..

[15] Wu M.Y., Gong H.Z., Hu K.R., Zheng H.Y., Wan X., Li J. (2021). Effect of syphilis infection on HIV acquisition: A systematic review and meta-analysis. Sex. Transm. Infect..

[16] Begovac J., Romih Pintar V., Vrsaljko N., Močibob L., Bogdanić N., Zekan Š., Đaković Rode O. (2023). Incidence, risk factors, and clinical findings of syphilis among men living with HIV in Croatia during the COVID-19 pandemic. Sci. Rep..

[17] Lőrincz K., Meznerics F.A., Jobbágy A., Kiss N., Madarász M., Belvon L., Tóth B., Tamási B., Wikonkál N.M., Marschalkó M. (2022). STIs during the COVID-19 Pandemic in Hungary: Gonorrhea as a Potential Indicator of Sexual Behavior. Int. J. Environ. Res. Public Health.

[18] Berzkalns A., Thibault C.S., Barbee L.A., Golden M.R., Khosropour C., Kerani R.P. (2021). Decreases in Reported Sexually Transmitted Infections During the Time of COVID-19 in King County, WA: Decreased Transmission or Screening?. Sex. Transm. Dis..

[19] Steffen R., Lautenschlager S., Fehr J. (2020). Travel restrictions and lockdown during the COVID-19 pandemic—impact on notified infectious diseases in Switzerland. J. Travel Med..

[20] Pagaoa M., Grey J., Torrone E., Kreisel K., Stenger M., Weinstock H. (2021). Trends in Nationally Notifiable Sexually Transmitted Disease Case Reports During the US COVID-19 Pandemic, January to December 2020. Sex. Transm. Dis..

[21] Soriano V., Blasco-Fontecilla H., Gallego L., Fernández-Montero J.V., de Mendoza C., Barreiro P. (2023). Rebound in sexually transmitted infections after the COVID-19 pandemic. AIDS Rev..

[22] Cusini M., Benardon S., Vidoni G., Brignolo L., Veraldi S., Mandolini P.L. (2021). Trend of main STIs during COVID-19 pandemic in Milan, Italy. Sex. Transm. Infect..

[23] Mitjà O., Padovese V., Folch C., Rossoni I., Marks M., Rodríguez I.A.M.A., Telenti A., Ciuffi A., Blondeel K., Mårdh O. (2023). Epidemiology and determinants of reemerging bacterial sexually transmitted infections (STIs) and emerging STIs in Europe. Lancet Reg. Health Eur..

[24] Tumalán-Gil O.D., Ruiz-González V., García-Cisneros S., González-Rodríguez A., Herrera-Ortiz A., Olamendi-Portugal M., Sánchez-Alemán M.A. (2023). High Incidence, Reinfections, and Active Syphilis in Populations Attending a Specialized HIV Clinic in Mexico, a Dynamic Cohort Study. Arch. Sex. Behav..

[25] Lee S.H., Lee J.E., Lee S.O., Lee S., Ko W.S., Kim H.H., Shin K.H., Kang J.S., Son H. (2024). Temporal Trends in Syphilis Incidence among Men with HIV in Busan, Korea, 2005–2022: A Retrospective Cohort Study. Viruses.

[26] Nazir A., Masood W., Ahmad S., Nair A.M., Aborode A.T., Khan H.D., Farid S., Raza M.A., Audah K.A. (2022). Rise of syphilis surge amidst COVID-19 pandemic in the USA: A neglected concern. Ann. Med. Surg..

[27] Vasalou V., Paparizos V., Daskalakis E., Paparizou E., Kourkounti S., Stratigos A. (2021). Syphilis and gonorrhoea among people with HIV during pandemic times: A report from Athens, Greece. Sex. Transm. Infect..

[28] Nessaibia I., Sagese R., Atwood L., Bouslama Z., Cocci L., Merad T., Tahraoui A. (2022). The way COVID-19 transforms our sexual lives. Int. J. Impot. Res..

[29] Shilo G., Mor Z. (2020). COVID-19 and the Changes in the Sexual Behavior of Men Who Have Sex With Men: Results of an Online Survey. J. Sex. Med..

[30] Sanchez T.H., Zlotorzynska M., Rai M., Baral S.D. (2020). Characterizing the Impact of COVID-19 on Men Who Have Sex with Men Across the United States in April, 2020. AIDS Behav..

[31] Bonato F., Ferreli C., Satta R., Rongioletti F., Atzori L. (2021). Syphilis and the COVID-19 pandemic: Did the lockdown stop risky sexual behavior?. Clin. Dermatol..

[32] D’Avanzo P.A., LoSchiavo C.E., Krause K.D., Karr A., Halkitis P.N. (2022). Biological, Behavioral, and Demographic Drivers of Recent Syphilis Infection Among Emerging Adult Sexual Minority Men in New York City: The P18 Cohort Study. AIDS Patient Care STDS.

[33] Stephenson R., Chavanduka T.M.D., Rosso M.T., Sullivan S.P., Pitter R.A., Hunter A.S., Rogers E. (2021). Sex in the Time of COVID-19: Results of an Online Survey of Gay, Bisexual and Other Men Who Have Sex with Men’s Experience of Sex and HIV Prevention During the US COVID-19 Epidemic. AIDS Behav..

