# Supplementary material for: The Resurgence of Treponema pallidum Infections and Reinfections during the COVID-19 Pandemic in Greece
Source: Int J Environ Res Public Health. 2024 Sep 26;21(10):1283. doi: 10.3390/ijerph21101283 (PMC11507046; doi:10.3390/ijerph21101283)
Supplement: Supplementary file 1 [file ijerph-21-01283-s001.zip › ijerph-3144701-supplementary.pdf]

Definition of infections

New infections were defined based on positive syphilis treponemal tests (TP-PA and/or EIA) with a previous negative test and/or no previous history of treated infection, and a four-fold increase in VDRL titer, whether in combination with clinical signs or not. Past infections were defined as those cases that responded to treatment with a ≥4-fold reduction in their VDRL titer, while at the time of testing had positive treponemal tests (TP-PA and/or EIA) and non-reactive VDRL test (or maintained the ≥4-fold reduced VDRL titer), and additionally did not develop any clinical signs of infection in the subsequent period. Last, past syphilis cases that responded to treatment with a ≥4-fold reduction in their VDRL titer, and upon hospital admission had a new increase (≥4-fold) in their VDRL titer and were recorded as re-infections [1–4].

1. Marchese, V.; Tiecco, G.; Storti, S.; Degli Antoni, M.; Calza, S.; Gulletta, M.; Viola, F.; Focà E.; Matteelli, A.; Castelli, F. Quiros-Roldan E. Syphilis Infections, Reinfections and Serological Response in a Large Italian Sexually Transmitted Disease Centre: A Monocentric Retrospective Study. *J. Clin. Med.* **2022**, *11*, 7499.
2. Boog, G.H.P.; Lopes, J.V.Z.; Mahler, J.V.; Solti, M.; Kawahara, L.T.; Teng, A.K.; Munhoz, J.V.T. Levin A.S. Diagnostic tools for neurosyphilis: A systematic review. *BMC Infect. Dis.* **2021**, *21*, 568.
3. Wu, M.Y.; Gong, H.Z.; Hu, K.R.; Zheng, H.Y.; Wan, X.; Li, J. Effect of syphilis infection on HIV acquisition: a systematic review and meta-analysis. *Sex. Transm. Infect.* **2021**, *97*, 525–533.
4. Begovac, J.; Romih Pintar, V.; Vrsaljko, N.; Močibob, L.; Bogdanić, N.; Zekan, Š.; Đaković Rode, O. Incidence, risk factors, and clinical findings of syphilis among men living with HIV in Croatia during the COVID-19 pandemic. *Sci. Rep.* **2023**, *13*, 11784.
